# Supplementary material for: Prevalence of diabetic foot at risk of ulcer development and its components stratification according to the international working group on the diabetic foot (IWGDF): A systematic review with metanalysis
Source: PLoS One. 2023 Nov 28;18(11):e0284054. doi: 10.1371/journal.pone.0284054 (PMC10684108; doi:10.1371/journal.pone.0284054)
Supplement: S1 Table — (DOCX) [file pone.0284054.s002.docx]

**S1. Search strategy**

|  |  | Date | Results |
| --- | --- | --- | --- |
| Pubmed | #1  prevalence[mesh] OR “Mass Screening” [mesh] OR screen*[tiab] OR Cross-Sectional Studies[Mesh] OR “cross-sectional”[tiab] ORprevalenc*[tiab] OR transvers*[tiab]  #2  risk [mesh] OR “risk assessment” [mesh] OR risk[tiab] OR at-risk[tiab]  #3  Diabetic foot [mesh] OR Foot ulcer[mesh] OR “diabetic foot” [tiab] OR “foot ulcer” [tiab] OR“diabetic feet” [tiab]  #4  "International Working Group on the Diabetic Foot" [tiab] OR IWGDF [tiab] OR classificat* [tiab] OR stratificat* [tiab]  (#1 AND #2 AND #3 AND #4) | 4th of March 2022 | 125 |
| Scopus | #1  TITLE-ABS-KEY(prevalenc*OR screen* OR Cross$Sectional OR transvers*)  #2  TITLE-ABS-KEY(*risk)  #3  TITLE-ABS-KEY((diabet* W/2 (foot OR feet)) OR “foot ulcer”)  #4  TITLE-ABS-KEY("International Working Group on the Diabetic Foot" OR IWGDF OR classificat* OR stratificat*)  (#1 AND #2 AND #3 AND #4) |  | 62 |
| WOS | #1  TS=(prevalenc*OR screen* OR Cross$Sectional OR transvers*)  #2  TS=(*risk)  #3  TS=((diabet* NEAR/2 (foot OR feet)) OR “foot ulcer”)  #4  TS=("International Working Group on the Diabetic Foot" OR IWGDF OR classificat* OR stratificat*)  (#1 AND #2 AND #3 AND #4) |  | 261 |
| Embase | #1  (‘prevalence’ OR ‘screening' OR ‘cross-sectional study’)/exp OR (prevalenc*OR screen* OR Cross-Sectional OR ‘cross sectional’ OR transvers*):ti,ab,kw  #2  (risk OR at-risk):ti,ab,kw  #3  (‘diabetic foot’ OR ‘foot ulcer’)/exp OR ((diabet* NEAR/2 (foot OR feet)) OR ‘foot ulcer’):ti,ab,kw  #4  (‘International Working Group on the Diabetic Foot’ OR IWGDF OR classificat* OR stratificat*):ti,ab,kw  (#1 AND #2 AND #3 AND #4) |  | 291 |
