# Supplementary material for: Prevalence of diabetic foot at risk of ulcer development and its components stratification according to the international working group on the diabetic foot (IWGDF): A systematic review with metanalysis
Source: PLoS One. 2023 Nov 28;18(11):e0284054. doi: 10.1371/journal.pone.0284054 (PMC10684108; doi:10.1371/journal.pone.0284054)
Supplement: S2 Table — (DOCX) [file pone.0284054.s003.docx]

**S2 Table. Studies that were evaluated in full-text, and were excluded.**

|  | Author | Year | Title | Exclusion reason |
| --- | --- | --- | --- | --- |
| 1 | Abrao | 2009 | Sensitivity of the feet to the 5.07 semmes-weinstein monofilament: Equivalence of results with use of 10 and 3 foot testing sites | Summary |
| 2 | Acquati | 2010 | Diabetic foot screening: An observational study in a population of diabetics in Forli' (Northern Italy) | Summary |
| 3 | Banik | 2016 | Discordance of standard classification systems to detect the risk of foot ulcer among type 2 diabetic subjects | Summary |
| 4 | De Almeida | 2015 | Prevalence and determinants of diabetic polyneuropathy and diabetic foot complications in a specialized clinic | Summary |
| 5 | De Corrado | 2013 | A multidisciplinary foot care team approach can lower the incidence of diabetic foot ulcers and amputation: Results of the asti study at 12 years | No Access to full text |
| 6 | Ferreira | 2019 | Foot function and strength of patients with diabetes grouped by ulcer risk classification (IWGDF) | Incomplete stratification |
| 7 | Hemmati | 2021 | Beyond diabetes mellitus type 2: Neuropathy, arterial disease and foot deformity | No Access to full text |
| 8 | Lee | 2021 | Risk assessment and classification for foot ulceration among patients with type 2 diabetes in South Korea | No Access to full text |
| 9 | Mineoka | 2017 | Relationship between limited joint mobility of the hand and diabetic foot risk in patients with type 2 diabetes | Incomplete stratification |
| 10 | Mineoka | 2019 | Platelet to lymphocyte ratio correlates with diabetic foot risk and foot ulcer in patients with type 2 diabetes | Same population |
| 11 | Monteiro | 2018 | Vascular and neurological evaluation of foots the people with diabetes mellitus type 2 | Summary |
| 12 | Monteiro-Soares | 2011 | Diabetic foot ulcer risk stratification systems: Which one to choose? A validation study | Summary |
| 13 | Nobili | 2017 | Extensive screening for ulcerative risk confirms the high prevalence of foot pathology and its role as marker of comorbidity in general diabetic population | Summary |
| 14 | Richard | 2014 | Screening patients at risk for diabetic foot ulceration: A comparison between measurement of vibration perception threshold and 10-g monofilament test | Incomplete stratification |
| 15 | Sandi | 2020 | Evaluation risk of diabetic foot ulcers (DFUs) using infrared thermography based on mobile phone as advanced risk assessment tool in the community setting: A multisite cross-sectional study | Incomplete stratification |
| 16 | Tomita | 2015 | Development and assessment of a simple scoring system for the risk of developing diabetic foot | Case control study |
| 17 | Stotl | 2022 | Individualised screening of diabetic foot: creation of a prediction model based on penalised regression and assessment of theoretical efficacy | No Access to full text |
| 18 | Zuhir | 2011 | Foot risk assessment in diabetic patients. An audit which explores the efficiency of foot risk assessment in patients' with diabetes | Summary |
| 19 | Santos | 2015 | Diabetic foot risk analysis in an interdisciplinary diabetes clinic | IWGDF non-classification |
| 20 | Lavery | 2007 | Reevaluating the Way We Classify the Diabetic Foot | Same population |
| 21 | Moura | 2013 | Risk factors for ulceration and amputation in diabetic foot: study  in a cohort of 496 patients | IWGDF non-classification |
| 22 | Wrobel | 2011 | Creating a diabetes foot reminder-based registry using the electronic medical record | Incomplete stratification |
