# Supplementary material for: Prevalence of diabetic foot at risk of ulcer development and its components stratification according to the international working group on the diabetic foot (IWGDF): A systematic review with metanalysis
Source: PLoS One. 2023 Nov 28;18(11):e0284054. doi: 10.1371/journal.pone.0284054 (PMC10684108; doi:10.1371/journal.pone.0284054)
Supplement: S3 Table — (DOCX) [file pone.0284054.s004.docx]

**S3.** **Evaluation of the quality of prevalence studies**

| **Evaluation of the quality of prevalence studies according to the Loney Scale for cross-sectional studies** | | | | | | | | | |
| --- | --- | --- | --- | --- | --- | --- | --- | --- | --- |
| **Study (Year)** | 1. Are the sampling design and method appropriate for the question? | 2. Is the sampling frame appropriate? | 3. Adequate sample size (>384 subjects) | 4. Are the criterio validated, objective and standardized to evaluate the disease? | 5. Was the outcome measured in an unbiased way (no bias)? | 6. Is the response rate adequate? Are those who reject described? (>70 response) | 7. Were estimates given with confidence intervals or subgroups? | 8. Is the target population described? | Total |
| Peters et. al. (2001) | NO | NO | NO | YES | YES | YES | YES | YES | 5 |
| Malgrange et.al. (2003) | NO | NO | YES | YES | YES | YES | YES | YES | 6 |
| Mugambi et.al. (2009) | NO | NO | NO | YES | YES | YES | YES | YES | 5 |
| González de la Torre et. al. (2010) | NO | NO | NO | YES | YES | YES | YES | YES | 5 |
| Monteiro-Soares et. al. (2012) | NO | NO | NO | YES | YES | YES | YES | YES | 5 |
| Shahbazian et. al.  (2013) | NO | NO | YES | YES | YES | YES | NO | YES | 5 |
| Bortoletto et. al. (2014) | NO | NO | NO | YES | YES | YES | YES | YES | 5 |
| Alonso-Fernandez et. al. (2014) | NO | NO | YES | YES | YES | YES | YES | YES | 6 |
| Tshitenge et. al. (2014) | NO | NO | NO | YES | YES | YES | YES | YES | 5 |
| Wu et. al. (2015) | NO | NO | NO | YES | YES | YES | YES | YES | 5 |
| Isip  (2016) | NO | NO | NO | YES | YES | YES | YES | YES | 5 |
| Damas-Casani et. al.  (2017) | NO | NO | NO | YES | YES | YES | YES | YES | 5 |
| Khan et. al. (2017) | NO | NO | NO | YES | YES | YES | YES | YES | 5 |
| Saraiva et. al. (2018) | NO | NO | NO | YES | YES | YES | YES | YES | 5 |
| Rodriguez et. al. (2018) | NO | NO | NO | YES | YES | YES | YES | YES | 5 |
| Vibha et. al. (2018) | NO | NO | YES | YES | YES | YES | YES | YES | 6 |
| Cardona et. al. (2018) | NO | NO | YES | YES | YES | YES | NO | YES | 5 |
| Tindong et. al. (2018) | NO | NO | NO | YES | YES | YES | YES | YES | 5 |
| Ramirez et. al. (2019) | NO | NO | NO | YES | YES | YES | NO | YES | 4 |
| Cardoso et. al. (2019) | NO | NO | NO | YES | YES | YES | YES | YES | 5 |
| Banik et. al. (2020) | NO | NO | YES | YES | YES | YES | YES | YES | 6 |
| Zantour et. al. (2020) | NO | NO | NO | YES | YES | YES | YES | YES | 5 |
| Gonzalez de la Torre et. al. (2020) | NO | NO | NO | YES | YES | YES | YES | YES | 5 |
| Mizouri et. al. (2021) | NO | NO | NO | YES | YES | YES | YES | YES | 5 |
| Castañeira et al.  (2018) | NO | NO | NO | YES | YES | YES | NO | YES | 4 |
| Mineoka et al.  (2022) | NO | NO | YES | YES | YES | YES | YES | YES | 6 |
| Formiga et al.  (2020) | NO | NO | NO | YES | YES | YES | NO | YES | 4 |
| Elshawary et al.  (2012) | NO | NO | NO | YES | YES | YES | YES | YES | 5 |
| Alvarez et al.  (2015) | NO | NO | NO | YES | YES | YES | YES | YES | 5 |
| Lavery et al.  (2003) | NO | NO | YES | YES | YES | YES | YES | YES | 6 |
| Ndip et al.  (2010) | NO | NO | NO | YES | YES | YES | YES | YES | 5 |
| Ndip et al.  (2010) | NO | NO | YES | YES | YES | YES | YES | YES | 6 |
| Yusuf et al.  (2016) | NO | NO | NO | YES | YES | YES | YES | YES | 5 |
| Doria et al.  (2016) | NO | NO | NO | YES | YES | YES | YES | YES | 5 |
| Bañuelos et al.  (2013) | NO | NO | NO | YES | YES | YES | NO | YES | 4 |
| Akila et al.  (2021) | NO | NO | NO | YES | YES | YES | YES | YES | 5 |
