# Supplementary material for: Prevalence of diabetic foot at risk of ulcer development and its components stratification according to the international working group on the diabetic foot (IWGDF): A systematic review with metanalysis
Source: PLoS One. 2023 Nov 28;18(11):e0284054. doi: 10.1371/journal.pone.0284054 (PMC10684108; doi:10.1371/journal.pone.0284054)
Supplement: Attachment — Submitted filename: Response PloS One.docx [file pone.0284054.s005.docx]

**Prevalence of diabetic foot at risk of ulcer development and its components stratification according to the International Working Group on the Diabetic Foot (IWGDF): A systematic review with metanalysis**

**COMMENTS**

The authors have done a good job by investigating the prevalence of diabetic foot at risk of ulcer development, which has contributed to knowledge by being the first study.

There are only very few typographical errors

1 – line 73: "y" should change to "and"

2 – line 80: "y" should also change to "and"

3 – line 115: (TMV y LFPM) should change to “(TMV and LFPM)”

4 – line 138: (TMV y LFPM) should change to “(TMV and LFPM)”

5- line 139: “synthetized” should be changed to “synthesized”

6 – line 242: “on Africa” should change to “in Africa”

7 – line 252: “prevalence de 44.3%” should change to “prevalence of	44.3%”

8 – line 401:” to stablish” should change to “to establish”
