# Supplementary material for: Prevalence of diabetic foot at risk of ulcer development and its components stratification according to the international working group on the diabetic foot (IWGDF): A systematic review with metanalysis
Source: PLoS One. 2023 Nov 28;18(11):e0284054. doi: 10.1371/journal.pone.0284054 (PMC10684108; doi:10.1371/journal.pone.0284054)
Supplement: Attachment — Submitted filename: Response PloS One v4.docx [file pone.0284054.s006.docx]

Dear reviewers, a response is given to each of the observations

Reviewer 1

COMMENTS

The authors have done a good job by investigating the prevalence of diabetic foot at risk of ulcer development, which has contributed to knowledge by being the first study.

There are only very few typographical errors

**1 – line 73: "y" should change to "and"**

Corrected

**2 – line 80: "y" should also change to "and"**

Corrected

**3 – line 115: (TMV y LFPM) should change to “(TMV and LFPM)”**

Corrected

**4 – line 138: (TMV y LFPM) should change to “(TMV and LFPM)”**

Corrected

**5- line 139: “synthetized” should be changed to “synthesized”**

Corrected

**6 – line 242: “on Africa” should change to “in Africa”**

Corrected

**7 – line 252: “prevalence de 44.3%” should change to “prevalence of	44.3%”**

Corrected

**8 – line 401:” to stablish” should change to “to establish”**

Corrected

ACADEMIC EDITOR

**introduction**

**Does the first sentence refer to worldwide estimates? To any diabetes?**

**I do not understand the sentence towards the end of para 1 " Moreover, the healthcare costs for diabetic foot treatment equals costs related to oncological fields, being a burden for developing countries " – what does that mean? please clarify with some statistical data?**

Answer

We modified the introduction according to the suggestions.

**Methods**

**The authors say that if there is significant heterogeneity they will conduct a subgroup analysis by continents, sex, type of study, type of DM, type of population, age group, time of DM and history of CKD was performed. What is the a priori justification for selecting these variables as potential sources of heterogeneity?**

Answer

We modified methods according to the suggestions.

**Results**

**In general, I found it hard to wade through the results. Virtually all numbers presented in tables are reiterated in the text - this is unnecessary and I would recommend only the relevant results are emphasised with numbers.**

**The data seem to show important regional differences e.g. prevalence rates seems to be very high in the South America and Central America (SACA) presented the highest prevalence with the lowest prevalence was identified on Africa compared to other regions. These are important findings that need discussion in terms of implications in practices and research in particular in limited resources countries in those regions.**

**It was virtually impossible to read the forest plans**

Answer

We modified the results according to the suggestions.

wWe improve the resolution of the figures

**Discussion**

**I request that authors expand the discussion to be more narrative and also forward-looking and also including a section on directions of future research.**

Answer

We modified the discussion according to the suggestions and added a paragraph on future research.

“Given the multiple forms of evaluation of neuropathy. Comparing possible versus probable neuropathy according to the Toronto Consensus in ulcer development is important. Likewise, we generally find 30% arterial calcification in the ABI that limits the determination of flow. Therefore, we must validate adding the evaluation of the arterial waveform by portable vascular Doppler for ulcer occurrence.

Carrying out studies based on the hospital population is more feasible. But we should know the frequency of foot at risk in the general population, where the prevalence is lower, and we find people with better diabetes control or less access to health services.”

**Conclusions**

**Weak conclusion which says little - can you say something more concrete**

Answer

We modified the conclusion according to the suggestions.

“The overall prevalence of foot at risk is high on worldwide. We identified high between-study heterogeneity and significant limitations (e.g limited countries, heterogeneous definitions, hospital-based studies, low certainty level of evidence). We need upgraded research using standardized and population-based studies and urgent action against preventable causes of diabetic foot.”

**Edriam Nim Tolentino**

**1. Please provide additional details regarding participant consent. In the Methods section, please ensure that you have specified (1) whether consent was informed and (2) what type you obtained (for instance, written or verbal). If your study included minors, state whether you obtained consent from parents or guardians. If the need for consent was waived by the ethics committee, please include this information**.

Our study was a systematic review of published studies. It was not required to obtain informed participation from people. However, we request authorization from an ethics committee authorized in Peru.

In the manuscript

“ This research did not include people, we only evaluated published studies. It was not necessary to require participan consent. We obtained authorization from the Institutional Ethics and Research Committee of thethrough the certificate 122-CIEI-CIENTIFICA-2021”

**2. We note that Figure 1 in your submission contain [map/satellite] images which may be copyrighted. All PLOS content is published under the Creative Commons Attribution License (CC BY 4.0), which means that the manuscript, images, and Supporting Information files will be freely available online, and any third party is permitted to access, download, copy, distribute, and use these materials in any way, even commercially, with proper attribution. For these reasons, we cannot publish previously copyrighted maps or satellite images created using proprietary data, such as Google software (Google Maps, Street View, and Earth). For more information, see our copyright guidelines:** [**http://journals.plos.org/plosone/s/licenses-and-copyright**](http://journals.plos.org/plosone/s/licenses-and-copyright)**.**

Figure 1 is an editable diagram of selection of included studies. The developers themselves require its use in systematic reviews. We place the citation and permission for its use granted by the developer.

*From:* Page MJ, McKenzie JE, Bossuyt PM, Boutron I, Hoffmann TC, Mulrow CD, et al. The PRISMA 2020 statement: an updated guideline for reporting systematic reviews. BMJ 2021;372:n71. doi: 10.1136/bmj.n71. The PRISMA Statement and the PRISMA Explanation and Elaboration document are distributed under the terms of the Creative Commons Attribution License, which permits unrestricted use, distribution, and reproduction in any medium, provided the original author and source are credited. (http://prisma-statement.org/PRISMAStatement/CitingAndUsingPRISMA.aspx

We replace figure 2 with another image with the requested license cc by 4.0

[“Continental organizations”](https://commons.wikimedia.org/wiki/File:Continental_organizations.svg#filelinks) by [Sbb1413](https://commons.wikimedia.org/wiki/User:Sbb1413) is licenced under [CC-BY 4.0](https://commons.wikimedia.org/wiki/Template:Cc-by_4.0) /Modified from original.
